# Health and wellbeing outcomes associated with loneliness for people with disability: a scoping review

**DOI:** 10.1186/s12889-023-17101-9

**Published:** 2023-11-29

**Authors:** Jodie Bailie, Glenda M. Bishop, Hannah Badland, Eric Emerson, Zoe Aitken, Roger Stancliffe, Kanchana Ekanayake, Gwynnyth Llewellyn

**Affiliations:** 1 Centre for Disability Research and Policy, The University of Sydney, Camperdown, NSW 2006 Australia; 2 University Centre for Rural Health, The University of Sydney, 61 Uralba Street, Lismore, NSW 2480 Australia; 3 Melbourne School of Population and Global Health, The University of Melbourne, Parkville, VIC 3010 Australia; 4 Social and Global Studies Centre, RMIT University, Melbourne, VIC 3000 Australia; 5 Centre for Disability Research, Faculty of Health & Medicine, Lancaster University, Lancaster, LA1 4YW UK; 6 University of Sydney Library, Camperdown, NSW 2006 Australia

**Keywords:** Loneliness, Disability, Health, Wellbeing, Scoping review

## Abstract

**Background:**

Loneliness is a significant public health concern due to its detrimental impact on health and wellbeing. Despite people with disability reporting higher levels of loneliness than the general population, there has been little research into how this is affecting their health and wellbeing. In light of this, the aim of our study was to scope both the existing evidence about the health and wellbeing outcomes associated with loneliness for people with disability, as well as the conceptual frameworks and measures utilised in this field of research.

**Methods:**

To conduct this scoping review, we followed the methodology outlined by JBI and searched MEDLINE, Scopus, Informit, Embase, and Web of Science for peer-reviewed, English-language articles published between 1 January 2000 and 8 February 2023. Two independent reviewers completed screening, full-text review and data extraction, with consensus sought at each stage. Data were analysed using content analysis and presented both numerically and narratively.

**Results:**

Out of the initial 1602 publications identified in the scoping review, only nine were included after duplicate removal, title and abstract screening, and full-text review. This limited number of studies, with the earliest study one published in 2015, represents a key finding. Eight of the nine studies were quantitative, and all were conducted in high income countries. Most of these studies utilised a version of the University of Los Angles Loneliness Scale to measure loneliness and addressed specific impairment groups. Notably, most of the studies identified associations between loneliness and health and wellbeing outcomes for people with disability.

**Conclusions:**

This scoping review highlights the current scarcity of studies examining the effect that loneliness has on the health and wellbeing outcomes of people with disability. As most of the reviewed studies relied on loneliness measures designed for individuals without disability, they potentially overlook the unique life experiences of people with disability. Given that loneliness is an international public health concern, it is imperative that people with disability are not left behind or overlooked in efforts to address the impact of loneliness on health and wellbeing.

**Supplementary Information:**

The online version contains supplementary material available at 10.1186/s12889-023-17101-9.

## Background

Meaningful social connections are essential for humans to thrive. Loneliness, defined as a “*a subjective unpleasant or distressing feeling of a lack of connection to other people, along with a desire for more, or more satisfying, social relationships”* [1], is closely linked to the quality of social connections as opposed to the quantity [2]. Globally, there is a growing concern about the rates and health consequences of loneliness [1, 3–6], with it now considered a public health priority [7, 8].

As noted in the editorial associated with this loneliness special issue, people with the greatest social disadvantage and marginalisation may have the highest rates of loneliness [8]. People with disability are one such vulnerable group. We know from previous research that they are more likely to report being lonely compared to those without disability [9–11]. For instance, analysis of data collected between 2016 and 2019 from the *English Community Life* survey, a nationally representative sample of approximately 17,000 adults, found that people with disability were over three times more likely to report feelings of loneliness than their peers without disability [9]. In a further study, Emerson and colleagues (2021) drew on the 2016–2019 waves of the United Kingdom (UK) *Understanding Society* survey, a nationally representative sample of approximately 35,000 adults, and found that adults with persistent disability (not just disability at some point in time) were over five times more likely to report ‘substantial’ loneliness than those without disability [10]. In both studies, disability is identified in the surveys by an affirmative response to two questions: the first asking about physical or mental health conditions or illnesses lasting or expected to last for 12 months or more and the second asking about whether the condition or illness result in difficulties carrying out day-to-day activities [9, 10].

People with disability are not a homogeneous group, for example it is likely that loneliness may be experienced quite differently by someone with severe intellectual disability and someone with a physical impairment associated with spinal cord injury. Some studies have suggested the degree of loneliness may be related to impairment. For example, an Australian study using data from the 2019 *Household, Income and Labour Dynamics in Australia (HILDA) Survey* found that working age people with psychosocial disability (47%) were most likely to experience loneliness while people with sensory disability (27%) were least likely to experience loneliness [12]. Stancliffe and colleagues (2010) in a study of over 13,000 users of intellectual and developmental disability services from 26 states in America found loneliness to be a widespread issue with 46% of all respondents reporting feeling lonely sometimes or often [13].

General population studies have shown that loneliness is associated with multiple adverse health outcomes, including a negative impact on mental health [6, 14–17], morbidity [17–19] and mortality [20, 21]. Given that people with disability are more likely to be at risk of loneliness, there is a high likelihood that they may also experience associated adverse health and wellbeing outcomes similar to or different from the general population. To the best of our knowledge there is no synthesis of the literature regarding the health and wellbeing outcomes associated with loneliness for people with disability.

Our primary aim was to scope the peer-reviewed published evidence about health and wellbeing outcomes associated with loneliness for ‘working age’ adults with disability (aged 15 – 64 years). Our second aim was to establish the conceptual frameworks and loneliness measures used by researchers studying this topic. Our third and final aim was to report the strengths, limitations, and gaps in the published literature.

## Methods

We employed a scoping review methodology guided by the work of JBI [22, 23] and Levac and colleagues [24] in this area. The review was conducted in accordance with an a priori protocol that has been published [25]. Reporting was guided by the Preferred Reporting Items for Systematic Reviews and Meta-Analyses Extension for Scoping Reviews (PRISMA-ScR) checklist [26]. Critical appraisal and risk of bias assessment of identified publications were not conducted, consistent with JBI methodology for scoping reviews.

### Stage 1: research question

The scoping review research questions were as follows:

1. What, if any, health and wellbeing outcomes are associated with loneliness for people with disability of working age?
2. What conceptual frameworks and measures are being used to examine health and wellbeing outcomes associated with loneliness for people with disability?
3. What are the strengths, limitations and gaps in the published literature?

### Stage 2: relevant literature identification

An initial search of MEDLINE and Google Scholar was conducted in January 2023 by the first author (JB) to identify relevant studies and generate a list of search terms. A full search strategy was developed for MEDLINE in consultation with an academic librarian (KE), and the senior author (GL) who has content expertise in the field of disability and loneliness (Table 1). To further ensure inclusivity we mapped our search results with references in recent publications about loneliness and disability. Embase, Informit, MEDLINE, Scopus and Web of Science were searched on 10 February 2023 (Additional file 1). The search was filtered to include peer-reviewed journal articles published in English between 1 January 2000 and 8 February 2023. The study also included hand searches of cited publications within eligible studies.

**Table 1:** Literature review search terms

**Ovid MEDLINE**	
1	exp Disabled Persons/
2	(disab* or handicap* or disabilit*).mp
3	1 or 2
4	Loneliness/
5	(Lonely or loneliness*).mp
6	4 or 5
7	“Health and Wellbeing”.mp
8	(Well-being or welbeing or wellbeing).mp
9	health/ or mental health/
10	Health*.mp
11	7 or 8 or 9 or 10
12	3 and 6 and 11
13	limit 12 to yr = ”2000 -Current”

### Stage 3: study selection

Inclusion criteria were identified and refined by the review team, according to the schema set out by JBI as follows.

#### Population

People with disability, aged 15–64 years, defined as having a long-term impairment or health condition lasting more than six months (including episodic conditions such as mental illness) that is associated with an activity limitation or participation restriction. We excluded studies in which the age of the participants was not given, or in which a sub-group of adults up to the age of 64 years could not be differentiated. The population of interest was those of school leaving age and prior to retirement to focus attention on adults of working age with lifelong or acquired disability and to exclude disability associated with the ageing process.

#### Concepts

Our focus was on studies examining the association between loneliness and health and wellbeing outcomes, including both the physical and psychological aspects. Studies that focused on social exclusion or social isolation, rather than loneliness, were excluded as these terms are considered conceptually different in the loneliness literature. We also excluded studies where the primary purpose was to examine, at a point in time, the impact of the COVID-19 pandemic on aspects of life for people with disability.

#### Context

All countries and settings.

#### Types of evidence sources

Original empirical research including quantitative, qualitative, mixed-methods study designs, and reviews (scoping, narrative, systematic and meta-analytical) published in English in a peer-reviewed journal. The index year of 2000 was selected to capture more than 20 years of publications. If the full-text version of a publication was not available, we contacted the corresponding authors to request one; if it was still not forthcoming, the publication was excluded.

The search results were exported to Endnote v.X9 [27], and duplicates removed before importing to Covidence (Veritas Health Innovation, Melbourne) [28] for screening. Following a pilot test, two independent reviewers (JB, GL) conducted title and abstract screening using the pre-defined inclusion and exclusion criteria. The same two reviewers independently completed full-text screening of abstracts and, subsequently, full-text review of included articles. Any discrepancies were then able to be resolved through discussion.

### Stage 4: data extraction (data charting)

A data-charting tool aligned with the review questions, developed by JB and GL and inserted into Covidence, was used to extract information from eligible publications. The tool was developed a priori and piloted on four randomly selected studies, then refined through discussion and updated accordingly. No further changes were made to the data-extraction tool after piloting. Two reviewers (JB and GL) independently extracted data, then discussed and cross-checked their data extraction. In cases where extracted data differed between reviewers, consensus was reached through discussion.

### Stage 5: data analysis and synthesis

Following JBI guidance, we conducted a qualitative content analysis [23, 29] on the extracted data using our first two research questions as the organising frame. Both reviewers (JB and GL) read and re-read the charted data plus revisited the source publications to review context as we independently worked through our analysis.

### Deviations from the protocol

We note several deviations from the study protocol. Firstly, two independent reviewers screened all the titles and abstracts, as opposed to only 20 per cent of them as outlined in the protocol. Secondly, we excluded studies that were undertaken during the COVID-19 pandemic following the lead of Taylor et al. (2023) [8] in this special edition. In their editorial they rightly point out that loneliness is a phenomenon that is worthy of study in and of itself, irrespective of unusual social circumstances such as those experienced during the pandemic.

## Results

### Search results and study selection

**Fig. 1:**
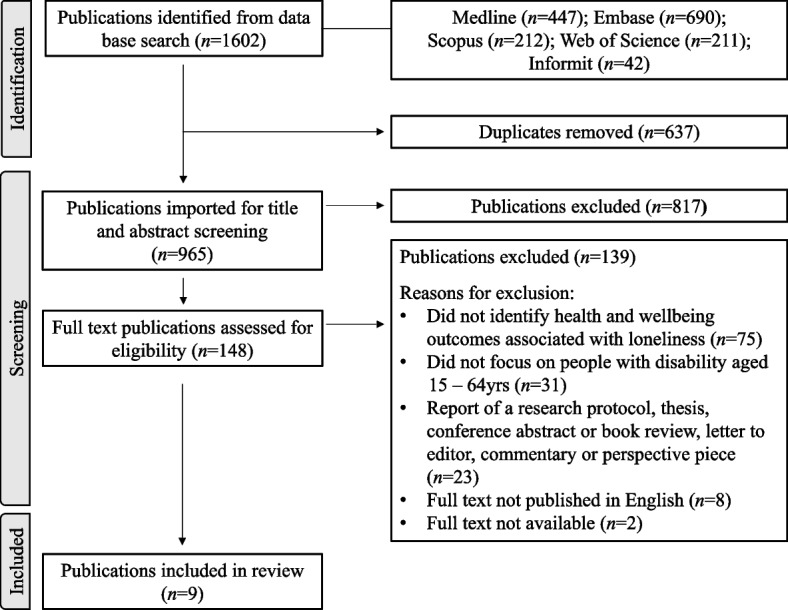
Preferred Reporting of Items for Systematic Reviews and Meta-Analysis Extension for Scoping Reviews (PRISMA-ScR) selection of sources of evidence flow diagram

The literature search identified 1602 publications. After duplicate removal, title and abstract screening, and full text review, nine publications were included for review (Fig. 1).

### Study country and year of publication

All of the studies in the nine reviewed publications were undertaken in high-income countries [30], with the number of publications from each of the five countries listed here in descending order: UK (*n* = 4), United States of America (*n* = 2), Canada (*n* = 1), Taiwan (*n* = 1) and Switzerland (*n* = 1). These nine publications were all published between 2015 and 2022, with none published between 2000–2014. Table 2 presents a summary of the nine articles.

**Table 2:** Summary of included articles, by alphabetical order of first author

**First study author | Year**	**Study design | Recruitment | Country | Study dates**	**Disability study population and identification**	**Sample size**	**Loneliness definitions and measure**	**Health and wellbeing measurement**	**Key findings related to health and wellbeing outcomes associated with loneliness**
Balto J, et al. (2018) [31]	**Study design:** Quantitative – cross-sectional, comparative study, age 18–64 years Data collected as part of another study examining measures of aerobic and muscular fitness in persons with multiple sclerosis compared with healthy adults **Recruitment:** North American Research Committee on multiple sclerosis registry and author lab database **Country:** Illinois, United States of America **Study dates:** Not stated	Multiple sclerosis **Determination:** Registration on research register	**Sample size:** Multiple sclerosis *n* = 63; comparison *n* = 21	**Definition:** Perlman and Peplau (1981) [32] **Measurement:** Original UCLA-LS 20-Item Scale	Hospital Anxiety and Depression Scale [33]—Measures the frequency of anxiety and depression symptoms over the past 4 weeks Modified Fatigue Impact Scale [34]—Examines patients’ perceptions of the functional limitations that fatigue has caused over the past month Multiple Sclerosis Impact Scale-29 (MSIS-29) [35] - Provides a measure of the physical and psychological impact of multiple sclerosis from the patient’s perspective (i.e., Quality of Life)	Depression, fatigue (both cognitive and psychosocial), and psychological quality of life were correlates of loneliness for people with multiple sclerosis Anxiety, physical fatigue, physical quality of life was not associated with loneliness for people with multiple sclerosis
Chang Y, et al. (2019) [36] +	**Study design**: Quantitative – cross-sectional survey, comparative study, age 10–19 years Primary data collection: survey mailed, self-administered **Recruitment:** Via flyers, e.g., in mainstream high schools, autism groups and via social media **Country:** Taiwan **Study dates**: Not stated	Autism spectrum disorder **Determination:** Independent diagnosis of autism spectrum disorder by a registered psychiatrist	**Sample size:** Autism spectrum disorder *n* = 101; comparison* n* = 101	**Definition**: Not stated **Measurement:** UCLA-LS 3-Item Scale (Chinese version)	Beck Anxiety Inventory (Chinese version) [37]- Measure of anxiety severity	Loneliness was significantly associated with greater anxiety among adolescents with autism spectrum disorder
Emerson E, et al. (2021) [9]	**Study design:** Quantitative – cross-sectional design, comparative study, age 16–64 years Secondary analysis of population-level data from national survey English *Community Life Survey* Online completion of survey Data were combined across three rounds of the *Community Life Survey* **Country:** United Kingdom **Study dates:** 2016–2019	Disability as a composite group **Determination:** Positive response to two questions in *Community Life Survey*	**Sample:** Total n = 17,723 (2016–2019) Average prevalence of disability, 19.1% (2016–2019)	**Definition:** Hawkley and Cacioppo (2010) [38] **Measurement:** Single item developed by UK Office for National Statistics	Four indicators of personal wellbeing developed by the UK Office for National Statistics: Satisfaction, Worth, Happiness, Anxiety. [39]	Loneliness had a significantly greater association with poorer personal wellbeing outcomes of satisfaction, worth, happiness and anxiety for people with disability
Emerson E, et al. (2021) [10]	**Study design:** Quantitative – cross-sectional-design, comparative study, aged 16–64 years Secondary analysis of population-level data from United Kingdom’s main annual household panel study national survey, *Understanding Society* Computer-assisted personal interviewing and computer-assisted self-completion **Study location:** United Kingdom **Study dates:** Wave 8 (2016–2018) and Wave 9 (2017–2019) though loneliness data only available in Wave 9	Disability as a composite group. Created four-category variable (No disability; Disability offset; Disability onset; Persistent disability) **Determination:** Positive response to two questions in *Understanding Society* survey	**Sample:** Wave 9 – 34,959 individuals in total; 76.6% reported no disability	**Definition:** Hawkley and Cacioppo (2010) [38] **Measurement:** UCLA-LS 3-Item scale plus single item developed by UK Office for National Statistics	12-Item Short Form Survey (SF-12) [40]- Measure of mental and physical functioning over the past 4 weeks 12-Item General Health Questionnaire (GHQ-12) [41]- Measure for screening for the onset of common mental health problems associated with depression, anxiety, somatic symptoms and social dysfunction	Loneliness was associated with the incidence and prevalence of mental health problems, but not the prevalence of physical health problems, for people with disability
Papagavri K, et al. (2020) [42]	**Study design:** Quantitative – cross-sectional design, comparative study, aged 16 + years Data from the population-level survey,* Adult Psychiatric Morbidity Survey* Trained interviewers, face-to-face computer-assisted interviewing, and self-completion, also using a computer. Assistance provided where necessary **Study location:** England **Study dates**: 2014	Borderline intellectual impairment **Determination:** Intellectual functioning measured using the National Adult Reading Test. Those with IQ below 80 were identified as having borderline intellectual impairment	**Sample***:* Borderline intellectual impairment *n* = 671; comparison* n* = 6206	**Definition:** Perlman and Peplau (1981) [32] **Measurement:** One item from the Social Functioning Questionnaire	14-Item Warwick-Edinburgh Mental Wellbeing Scale (WEMWBS) [43]—Measure of mental and physical functioning over the past four weeks – a quality of life measure Clinical Interview Schedule Revised (CIS-R) [44]—Measure to identify common mental disorders (depression, generalised anxiety disorder, agoraphobia, any phobia and panic disorder) who had been diagnosed and treated in the last 12 months Self-harm and suicide – bespoke measure used to identify if participants had thought about suicide in the last week and the last 12 months Physical health—bespoke measure to identify if participants had suffered from any of the following chronic diseases in the last 12 months: asthma, cancer, epilepsy, diabetes and high blood pressure Self-reported health – bespoke measure using one item: “how is your health in general?”	Those with borderline intellectual functioning who reported feeling lonely had lower wellbeing, were more likely to have depression, generalised anxiety disorder, agoraphobia and any type of phobia in the last 12 months, and to report suicidal thoughts in the past week and last year than those with borderline intellectual impairment who were not lonely. Loneliness was also associated with chronic diseases and poor self- reported health
Robinson-Whelen S, et al. (2016) [45]	**Study design:** Quantitative – cross-sectional survey. Data drawn from an existing longitudinal cohort study. Participants originally recruited between 1973 and 2011 during initial post-injury rehabilitation in hospital. Current study includes participants who completed a follow-up survey, therefore 5–40 years follow-up **Study location**: United States of America **Study dates:** April 2014–June 2015 (for follow-up survey)	Spinal cord injury – level of injury represented by paraplegia versus tetraplegia **Determination:** Former patient at a spinal cord post injury rehabilitation centre	**Sample***: n* = 175 with 58% tetraplegia, 39% paraplegia	**Definition:** Perlman and Peplau (1981) [32] **Measurement:** UCLA-LS 3-Item Scale	Patient Health Questionnaire-2 (PHQ-2) [46]- Measure to identify individuals with elevated depressive symptoms 5-Item Satisfaction with Life Scale [47]—Measure to identify life satisfaction	Loneliness was significantly related to life satisfaction and depression for people with a spinal cord injury
Santino N, et al. (2022) [48]	**Study design:** Quantitative – cross sectional survey. Data collected as part of a larger study examining issues of social isolation and health in people with spinal cord injury or dysfunction. Telephone survey **Study location:** Toronto, Canada **Study dates:** Not stated	Spinal cord injury or dysfunction. Level of injury represented by paraplegia versus tetraplegia **Determination:** Former patient at spinal cord rehabilitation program	**Sample:*** n* = 170 with traumatic and non-traumatic with spinal cord injury or dysfunction	**Definition:** Perlman and Peplau (1981) [32] **Measurement:** UCLA-LS 3-Item Scale	11-Item Life Satisfaction Questionnaire (LiSat-11) [49]—Measure to identify life satisfaction	Significant bivariate correlations were found between loneliness and life satisfaction for people with a spinal cord injury or dysfunction
Smith B and Caddick N. (2015) [50]	**Study design:** Qualitative study. Aged 18 + years Primary data collection, semi-structured life story interview. Criterion based, purposive sampling strategy. Open letter in United Kingdom disability newsletters and Internet sites **Study location:** United Kingdom **Study dates:** Not stated	Spinal cord injury **Determination:** Registered as disabled by a United Kingdom Government authority	**Sample:*** n* = 20 with spinal cord injury, 14 lived in a care home and 6 lived in community. Sample size determined by data saturation	**Definition**: Not stated **Measurement:** Qualitative inductive analysis	Qualitative responses	A theme in this qualitative study was that loneliness was perceived to damage psychological wellbeing for people with a spinal cord injury
Tough H, et al. (2017) [51]	**Study design:** Quantitative – cross sectional survey, aged 30–65 years. Data from existing longitudinal cohort study, namely the community survey of the *Swiss Spinal Cord Injury Cohort Study*. Standardised telephone interviews and questionnaires (paper–pencil or online) **Study location***:* Switzerland **Study dates:** May 2015–January 2016	Spinal cord injury, traumatic and non-traumatic **Determination:** Registration on research register	**Sample:*** n* = 133 with spinal cord injury; *n* = 133 partners of the persons with spinal cord injury	**Definition:** Hawkley and Cacioppo (2010) [38] **Measurement:** UCLA-LS 3-Item	36-Item Short Form Health Survey (SF-36 version 1.0) [52]—Measure to identify vitality and mental health	For people with spinal cord injury who reported feeling lonely, this was associated with feelings of decreased vitality and poorer mental health for people with a spinal cord injury

+ The study by Chang et al. (2019) is included as most of the sample are aged 15 to 19 years (73.3%, compared to 26.7% for participants aged 10 to 14 years) and loneliness data is disaggregated by age groups including for 15 to 19 years

*Note*: *UCLA-LS* University of California Los Angeles, Loneliness Scale

### Study purpose

The stated aims or objectives of six of the nine studies focused on investigating the health and wellbeing outcomes associated with loneliness for people with disability [9, 10, 31, 42, 45, 48] (Additional file 2). The remaining three studies reported on health and wellbeing outcomes associated with loneliness in the context of other study aims or objectives [36, 50, 51].

### Study methods and data sources

All nine studies (eight quantitative and one qualitative) used a type of cross-sectional study design. Seven of the nine utilised pre-existing data sources, which included population-level data from three separate national surveys conducted in the UK [9, 10, 42]: the *English Adult Psychiatric Morbidity Survey*, *Understanding Society*, and the *Community Life Survey,* (*Understanding Society* is a longitudinal cohort study; Emerson et al*.* 2021 [10] utilised cross-sectional analysis of 2 waves only). Additionally, data from existing longitudinal cohort studies for people with specific impairments / health conditions were used in two studies [45, 51], while in the remaining two studies data were collected as part of larger projects [31, 48].

Primary data collection methods – semi-structured life story interviews [50], and a self-administered paper-based questionnaire [36] – were employed in two of the nine studies.

Five of the nine studies drew comparisons between the health and wellbeing outcomes of people with disability and those without [9, 10, 36, 42, 51].

### Disability study population

All studies addressed community-dwelling adults, with one exception: Smith and Caddick (2015) focused on those individuals living or who had lived in a care home within the previous six months.

The nine studies employed various ways to describe people with disability. Seven focused on specific impairment or diagnostic groups, including those with spinal cord injury [45, 48, 50, 51], autism spectrum disorder [36], multiple sclerosis [31], and borderline intellectual impairment [42]. In contrast, two studies focused on people with disability as a group without differentiating by impairment or disability type [9, 10].

Six different approaches to determining disability status were taken. Three studies relied on positive responses to one or more disability-related questions in a survey [9, 10, 42]. Two relied on self-reporting of disability along with either a confirmed diagnosis by a registered psychiatrist [36] or being officially registered as disabled by a government authority [50]. Of the remaining four studies, two confirmed disability status by registration on a disability research register [31, 51], while the other two used patient attendance at a clinic or rehabilitation centre [45, 48].

### Loneliness conceptual framework

Seven studies based their understanding of loneliness on one of two well-known conceptual frameworks about loneliness, with the remaining two publications not providing a conceptualisation of loneliness [36, 50]. The most frequently used conceptual framework was that developed by Perlman and Peplau [31, 32, 42, 45, 48]. This approach defines loneliness as the discrepancy between a person’s desired and actual social relationships. According to Perlman and colleagues, loneliness is characterised as “the unpleasant experience that occurs when a person’s network of social relations is deficient in some important way, either quantitatively or qualitatively” [32]. This conceptualisation emphasises the objective (and therefore potentially measurable) features of an individual’s social network that may contribute to feelings of loneliness. The remaining three studies [9, 10, 51] drew on the conceptual framework proposed by Hawkley and Cacioppo [38]. This framework, in contrast to the objective features approach by Perlman and colleagues, emphasises the subjective experience of loneliness. According to this perspective, loneliness exists when an individual perceives a lack of desired social connections, regardless of the quantity or quality of their social network.

### Loneliness measures

Six of the eight quantitative studies used a version of the University of California Los Angeles Loneliness Scale (UCLA-LS) (Table 3) as the measure of loneliness: four employed the UCLA-LS 3-Item version [9, 45, 48, 51], one the original UCLA-LS 20-Item version, [31] and the remaining study used the UCLA-LS 8-Item Chinese version [36].

**Table 3:** Measurement or instruments used to assess loneliness in eight (quantitative) studies

**Loneliness instrument**	**Loneliness measure**	**Number of studies**	**Question or item wording**
UCLA-LS	Short-form UCLA-LS 3-Item Scale [53]	Three studies [45, 48, 54]	1. How often do you feel that you lack companionship? 2. How often do you feel left out? 3. How often do you feel isolated from others?
	Original UCLA-LS 20-Item Scale [55]	One study [31]	Originally released in 1978 as a 20-Item scale
	Short-form UCLA-LS 8-Item Scale Chinese Version [56]	One study [36]	This Chinese version contains 8 items, including 2 positively worded items (Item 3: “I am an outgoing person,” and Item 6: “I can find companionship when I want it”), which are reverse scored
UCLA-LS 3-Item plus single-item	Short-form UCLA-LS 3-Item Scale [53] Single item from Office for National Statistics [57]	One study [10]	1.How often do you feel that you lack companionship? 2. How often do you feel left out? 3. How often do you feel isolated from others? Single item How often do you feel lonely?
Single item only	Office for National Statistics [57]	One study [9]	Single item How often do you feel lonely?
	Social Functioning Questionnaire [58]	One study [42]	Single item I feel lonely and isolated from other people

*UCLA-LS* University of California Los Angeles, Loneliness Scale

The two studies, conducted by Emerson and colleagues (2021) [9, 10], were dependent on the loneliness measure incorporated in national surveys designed by the Office of National Statistics, which asks the question, “How often do you feel lonely?” [57]. The first study used the UCLA-LS 3-Item in addition to the single Office of National Statistics item [9], while the second study only used the single Office of National Statistics loneliness item found in the *English Community Life Survey *[10].

One study used a single item from the Social Functioning Questionnaire: “I feel lonely and isolated from other people” [42]. In their qualitative study, Smith and Caddick (2015) [50] posed a range of open-ended questions, including, “Can you tell me about your life in the care home”, “Can you describe how it feels to be living in a care home”. The impact of loneliness on health and wellbeing outcomes were identified by thematic analysis of the qualitative responses.

### Health and wellbeing measures

There were 16 different approaches to assessing health and wellbeing outcomes in the eight quantitative studies (Table 2). Mental health and wellbeing outcomes measured included the following^1^ (i) anxiety [9, 31, 36, 42]; (ii) depression [31, 45]; (iii) suicidal ideation [42]; (iv) wellbeing [42]; (v) life satisfaction [9, 45, 48]; (vi) vitality [51]; (vii) worth [9]; and (viii) happiness [9]. Physical health outcomes reported included functional limitations as a result of fatigue [31], self-reporting of health status [42], and identification of a chronic disease in the past 12 months [42]. The qualitative study used an inductive thematic analysis approach to identify the themes related to the health and wellbeing outcomes caused by loneliness for people with disability [50].

### Association between loneliness and health and wellbeing for people with disability

In Table 4 we present a summary of the identified associations between loneliness and health and wellbeing outcomes for people with disability, according to the measures used in each study. Additional file 3 provides extracts from studies on health and wellbeing outcomes.

**Table 4:** Health and wellbeing outcomes associated with loneliness for people with disability

**Outcomes**	**Health and wellbeing**	**Association with loneliness according to measures used in each study**	
		**Yes**	**No**
**Mental health and wellbeing outcomes**	Anxiety	3 studies [9, 36, 42]	1 study [31]
	Depression	3 studies [31, 42, 45]	
	Poorer overall mental health	2 studies [10, 51]	
	Reduced life satisfaction	3 studies [9, 45, 48]	
	Decreased wellbeing	2 studies [42, 50]	
	Cognitive fatigue	1 study [31]	
	Psychosocial fatigue	1 study [31]	
	Diminished feelings of happiness	1 study [9]	
	Diminished feelings of worth	1 study [9]	
	Diminished feelings of vitality	1 study [51]	
	Agoraphobia, and any type of phobia	1 study [42]	
	Suicidal ideation	1 study [42]	
	Lower psychological quality of life	1 study [31]	
**Physical health**	Poorer self-reported poor health	1 study [42]	
	Higher chronic disease rates	1 study [42]	
	Poorer physical health		1 study [10]
	Physical fatigue		1 study [31]
	Decreased physical quality of life		1 study [31]

## Discussion

### Main findings of this study

In this scoping review covering more than two decades of literature, we only identified nine articles that examined the association between loneliness and health and wellbeing outcomes for people with disability. Key findings from this scoping review suggest that research on this topic is just beginning to emerge, with the earliest study only published in 2015. Our findings demonstrate that: (i) relatively few studies have examined whether loneliness is associated with health and wellbeing (including adverse outcomes) for people with disability; (ii) even fewer studies use a comparison group of people without disability; (iii) the studies examine a wide range of health and wellbeing outcomes, which limits the conclusions that can be drawn from them; (iv) most studies continue to focus on people from specific impairment groups rather than from the broader population of people with disability; and (v) the studies predominantly rely on self-reported items about loneliness and a small suite of loneliness measures that, to the best of our knowledge, have not been evaluated for their validity in assessing the loneliness experienced by people with disability. Of note, all the studies came from high-income countries.

That said, our findings suggest that loneliness for people with disability is associated with poorer health and wellbeing, specifically mental health, but also poorer physical health in the studies where it has been possible to examine this outcome. These findings provide the impetus to develop our program of research further, with the aim of extending it beyond prevalence and correlational studies to incorporate study designs that examine the directionality of the association between poorer health and wellbeing outcomes and loneliness, and possible causality. Fried et al. (2020) [4] have also drawn attention to the need for more research into the impacts of loneliness on health and wellbeing outcomes for the general population.

All the studies reviewed used cross-sectional study designs. This highlights the need for longitudinal studies that examine this relationship between loneliness and health and wellbeing outcomes for people with disability and whether the association has persisted over time. As no low and middle-income country studies were included in this review there is a need for disability, loneliness and health and wellbeing research in these contexts.

The extant literature on loneliness and health and wellbeing outcomes for people with disability is currently limited to the two major conceptual approaches found for the general population and associated instruments to measure loneliness. This leaves a significant gap in understanding loneliness for people with disability and association with or impact on their health and wellbeing. There are two major concerns: the first relates to the loneliness measures used; and the second to the possible interactions between loneliness and health and wellbeing for people with disability.

Firstly, understanding the lived experience of loneliness for people with disability needs attention. As with other reviews that have examined loneliness measures, we found the UCLA-LS to be by far the most frequently used [59, 60]. It may be the case, as in other life areas, that the loneliness people with disability experience differs somewhat from those of their peers without disability, e.g., in the frequently experienced discrimination in public places [61]. If this is the case, it may be that the UCLA-LS and other measures currently used, although capable of uncovering significant differences in the prevalence of loneliness between people with disability and those without, may need to be revised or expanded to capture the nature and full extent of the loneliness experienced by people with disability. Similar to Gomez-Zuniga and colleagues (2023), we identified a lack of qualitative studies that explore the subjective experience of loneliness for people with disability. This is a major shortcoming given that loneliness is typically defined as a *subjective* unpleasant or distressing feeling [60].

Secondly, the loneliness literature for the general population suggests that loneliness and health have a bi-directional and cyclical relationship, meaning that each can influence the other [62, 63]. While this may also be the case for people with disability, there may be other factors at play. It is well established that people with disability experience inequalities in health care, resulting in poorer physical and mental health outcomes than people without disability [64–66]. This is especially important when considering associations between loneliness and health and wellbeing outcomes given that the increased prevalence of both issues could simply be due to the experience of disability, rather than loneliness promoting certain health and wellbeing outcomes or that certain health and wellbeing outcomes promote loneliness. It could be, for example, as the Emerson et al. 2021 [10] study included in this review demonstrated, that the association between loneliness and poorer health and wellbeing outcomes occurs only with persistent disability, potentially suggesting a cyclical relationship. It is still unclear from the literature whether this is the case for people with disability, but given the higher prevalence of loneliness for people with disability it does warrant further examination in the interests of promoting better health and wellbeing outcomes.

## Strengths and limitations

The strengths of our scoping review are: 1) a published a priori protocol [25] that improves the transparency and reproducibility of the scoping review; 2) the rigorous process of two reviewers independently conducting title and abstract screening, full text review and data extraction, and engaging in robust discussions to reach consensus at every stage; and 3) a search strategy designed with both an experienced academic librarian (KE) and a well-qualified research team.

Review limitations include: 1) the risk of language bias as only publications in English were included; 2) potentially missing relevant evidence as we excluded grey literature, including theses.; and 3) potentially missing relevant studies if they focused on specific impairment groups and did not use the terms ‘disability’ or ‘impairment’.

## Conclusions

This scoping review highlights the nascent state of research on the health and wellbeing outcomes associated with loneliness for people with disability. The scarcity of studies, the lack of comparison groups of people without disability, the reliance on measures designed for individuals without disability, and the narrow focus on specific impairments pose challenges to a comprehensive understanding of the topic. This is regrettable given the rise in loneliness reported in many countries and the ongoing impact of the isolation from their fellows that many people experienced during the COVID-19 pandemic. Work in several countries including Australia is attempting to understand the drivers of loneliness as COVID-19 and post-COVID 19 population data becomes available. It will be important that this work includes consideration of people with disability to understand the nature and extent of the impact of loneliness on their health and wellbeing and to ensure that they are not overlooked or left behind in any public health interventions for loneliness for the general population.

### Supplementary Information

**Additional file 1. **Search terms.

**Additional file 2.** Aims and objectives of included studies.

**Additional file 3.** Health and wellbeing associations with loneliness identified in studies.

## Data Availability

All data and materials generated or analysed are included in this article.

## Footnotes

1 The wellbeing measure used in the study by Emerson et al. (2021) has four components that are measured separately – satisfaction, anxiety, happiness and worth. We report these separately to align with the study publication.
