# Supplementary material for: Health and wellbeing outcomes associated with loneliness for people with disability: a scoping review
Source: BMC Public Health. 2023 Nov 29;23:2361. doi: 10.1186/s12889-023-17101-9 (PMC10685646; doi:10.1186/s12889-023-17101-9)
Supplement: Supplementary file 1 — Additional file 1. Search terms. [file 12889_2023_17101_MOESM1_ESM.docx]

**Additional file 1: Search terms**

**Ovid MEDLINE(R) ALL <1946 to February 08, 2023>**

1 exp Disabled Persons/ 72644

2 (disab* or handicap* or disabilit*).mp. 409741

3 1 or 2 423805

4 Loneliness/ 5938

5 (Lonely or loneliness*).mp. 13401

6 4 or 5 13401

7 "Health and Wellbeing".mp. 6465

8 (Well-being or welbeing or wellbeing).mp. 133250

9 health/ or mental health/ 82826

10 Health*.mp. 4519740

11 7 or 8 or 9 or 10 4563777

12 3 and 6 and 11 495

13 limit 12 to yr="2000 -Current" 447

**Embase <1974 to 2023 February 08>**

1 exp disabled person/ 64585

2 (disab* or handicap* or disabilit*).mp. 516357

3 1 or 2 528334

4 loneliness/ 13527

5 (Lonely or loneliness*).mp. 18069

6 4 or 5 18069

7 wellbeing/ 83928

8 "Health and Wellbeing".mp. 8291

9 (Well-being or welbeing or wellbeing).mp. 203193

10 health/ or mental health/ 427070

11 Health*.mp. 6385546

12 7 or 8 or 9 or 10 or 11 6454124

13 3 and 6 and 12 744

14 limit 13 to yr="2000 -Current" 690

**Informit**

20 of 33 results for [All Fields:lonely OR All Fields:loneliness*] AND All Fields:Health* AND All Fields:Disab* AND Publication Date: (01/01/2000 TO 08/02/2023)

1 – 9 of 9 results for[All Fields:lonely OR All Fields:loneliness*] AND All Fields:wellbeing AND All Fields:Disab* AND Publication Date: (01/01/2000 TO 08/02/2023)

**Scopus via Elsevier coverage-1996 to present**

212 document results

( TITLE-ABS-KEY ( disab* OR handicap* OR disabilit* ) AND TITLE-ABS-KEY ( lonely OR loneliness* ) AND TITLE-ABS-KEY ( "Health and Wellbeing" OR well-being OR welbeing OR wellbeing ) ) AND ( LIMIT-TO ( PUBYEAR , 2023 ) OR LIMIT-TO ( PUBYEAR , 2022 ) OR LIMIT-TO ( PUBYEAR , 2021 ) OR LIMIT-TO ( PUBYEAR , 2020 ) OR LIMIT-TO ( PUBYEAR , 2019 ) OR LIMIT-TO ( PUBYEAR , 2018 ) OR LIMIT-TO ( PUBYEAR , 2017 ) OR LIMIT-TO ( PUBYEAR , 2016 ) OR LIMIT-TO ( PUBYEAR , 2015 ) OR LIMIT-TO ( PUBYEAR , 2014 ) OR LIMIT-TO ( PUBYEAR , 2013 ) OR LIMIT-TO ( PUBYEAR , 2012 ) OR LIMIT-TO ( PUBYEAR , 2011 ) OR LIMIT-TO ( PUBYEAR , 2010 ) OR LIMIT-TO ( PUBYEAR , 2009 ) OR LIMIT-TO ( PUBYEAR , 2008 ) OR LIMIT-TO ( PUBYEAR , 2007 ) OR LIMIT-TO ( PUBYEAR , 2006 ) OR LIMIT-TO ( PUBYEAR , 2005 ) OR LIMIT-TO ( PUBYEAR , 2004 ) OR LIMIT-TO ( PUBYEAR , 2003 ) OR LIMIT-TO ( PUBYEAR , 2002 ) OR LIMIT-TO ( PUBYEAR , 2001 ) OR LIMIT-TO ( PUBYEAR , 2000 ) )

**Web of Science via Clarivate – coverage 1900 to present**

211 Documents

disab* OR handicap* OR disabilit* (Topic) and lonely OR loneliness* (Topic) and "Health and Wellbeing" OR well-being OR welbeing OR wellbeing (Topic) and 2000 or 2001 or 2002 or 2003 or 2004 or 2005 or 2006 or 2007 or 2008 or 2009 or 2010 or 2011 or 2012 or 2013 or 2014 or 2015 or 2016 or 2017 or 2018 or 2019 or 2020 or 2021 or 2022 or 2023 (Publication Years)
