# Supplementary material for: Health and wellbeing outcomes associated with loneliness for people with disability: a scoping review
Source: BMC Public Health. 2023 Nov 29;23:2361. doi: 10.1186/s12889-023-17101-9 (PMC10685646; doi:10.1186/s12889-023-17101-9)
Supplement: Supplementary file 2 — Additional file 2. Aims and objectives of included studies. [file 12889_2023_17101_MOESM2_ESM.docx]

**Additional file 2: Aims or objectives of included studies**

*Table 2.1: Aims or objectives of included studies, alphabetically by study authors*

| **First study author and year** | **Aim or objectives of included studies** |
| --- | --- |
| **Health and wellbeing outcomes associated with loneliness are reported in the study and stated in study aims or objectives.** | |
| Balto J, et al. (2018) (31) | “The specific aims were to (1) compare the extent of loneliness between persons with MS and healthy adults; (2) examine the association of sociodemographic variables, disability, and functional limitations and loneliness in persons with MS; and (3) examine depression, anxiety, fatigue, and quality of life as possible correlates of loneliness.” (Page 53) |
| Emerson E, et al. (2021)(9) | *“*To examine exposure to three indicators of low social connectedness (loneliness, low perceived social support, social isolation), and to evaluate the association between low social connectedness and wellbeing. To test whether disability status moderated the relationship between low social connectedness and wellbeing.” (Page 1) |
| Emerson E, et al. (2021)(10) | “… our aims were: i. To estimate the prevalence of exposure to loneliness among a nationally representative sample of adults in the UK with persistent disability, disability onset, disability offset and no disability (age range 16–64); ii. To identify personal demographic characteristics and aspects of living circumstances that may be associated with variation in exposure rates; iii. To investigate the association between loneliness and self-rated health and mental health among respondents with persistent disability, disability onset, disability offset and no disability.” (Page 534) |
| Papagavriel K, et al. (2020) (42) | “Our aim was to examine the prevalence of loneliness and the association between loneliness and socio-demographic and health outcomes in people with borderline intellectual impairment (BII) and the general population. Our objectives were to: 1. Compare the prevalence of loneliness in people with borderline intellectual impairment and the general population. 2. Explore associations between loneliness and age, sex, ethnicity, marital status, income, employment, social support, discrimination, and neighbourhood characteristics in both groups 3. Explore differences in the relationship between loneliness and common chronic diseases, self-reported physical health, mental wellbeing, common mental disorders, and suicidal thoughts in both groups 4. Explore interaction effects between level of intellectual functioning (people with borderline intellectual impairment and the general population) and loneliness in relation to the above socio-demographic and clinical variables.” (Page 955) |
| Robinson-Whelen S, et al. (2016)(45) | *“*To (1) examine a measure of loneliness and its correlates in people with spinal cord injury (SCI) to enhance our understanding of loneliness, which has received limited scientific study in the context of SCI; and (2) conduct preliminary analyses of the reliability and validity of the measure, including an evaluation of the unique impact of loneliness on psychological health.” (Page 1728) |
| Santino N, et al. (2022)(48) | “…the objectives of the present study were to: (a) assess the relationship between LTPA and loneliness among people with SCI/D, and (b) examine loneliness as a possible mediator of the relationship between LTPA and life satisfaction among individuals with SCI/D.” (Page 174) |
| **Health and wellbeing outcomes associated with loneliness are reported in the study, though not mentioned in the study aims or objectives.** | |
| Chang Y, et al. (2019)(36) | “This study compared the perceptions of adolescents with ASD with those of their neuro-typical peers toward their friendship quality, activity participation, and emotional well-being, and examined the relationships between friendship quality, activity participation, and emotional well-being.” (Page 452) |
| Smith B & Caddick N. (2015)(50) | This study examined the impact of living in a care home on health and wellbeing of people with spinal cord injury. |
| Tough H, et al. (2017)(51) | “To investigate 1) the variation in the quantity and quality of social relationships in persons with spinal cord injury (SCI) and their partners; 2) dyadic coherence within social relationship constructs; 3) the interrelationships between social relationship constructs; and 4) the associations of social relationship constructs with vitality and mental health.” (Page 294) |
