# Supplementary material for: Health and wellbeing outcomes associated with loneliness for people with disability: a scoping review
Source: BMC Public Health. 2023 Nov 29;23:2361. doi: 10.1186/s12889-023-17101-9 (PMC10685646; doi:10.1186/s12889-023-17101-9)
Supplement: Supplementary file 3 — Additional file 3. Health and wellbeing associations with loneliness identified in studies. [file 12889_2023_17101_MOESM3_ESM.docx]

**Additional file 3: Health and wellbeing associations with loneliness identified in studies**

*Table 3.1: Health and wellbeing association with loneliness identified in studies, alphabetically by study author*

| **First study author and year** | **Findings from included studies** |
| --- | --- |
| Balto J, et al. (2018)(31) | “There were statistically significant positive correlations in the MS sample between loneliness and depression (r= .49, p < .00), cognitive fatigue (r= .34, p < .01), and psychosocial fatigue (r= .30, p < .02). Loneliness was further associated with psychological QOL (r= .44, p < .00.” (Page 56)  “… and (3) depression, fatigue, and QOL represented correlates of loneliness among those with MS” (Page 56)  Table 3 shows no association between anxiety, physical fatigue, physical quality of life. (Page 57) |
| Chang Y, et al. (2019)(36) | “Loneliness was significantly correlated with friendship quality and anxiety (see Table 2); thus, a mediation effect (where the mediator predicts the outcome) would be supported by a finding that the significant relationship between friendship quality and anxiety was reduced or became nonsignificant relationship between friendship quality and anxiety after entering the mediator in the regression models.” (Page 457)  “Figure 3 confirms that loneliness mediated the relationship between friendship quality and anxiety, and based on analyzes using Sobel's test, the mediation effect was found to be significant. Figure 3 also indicates that the greater anxiety in participants with lower friendship quality was accounted for by their higher level of loneliness.” (Page 457)  “… loneliness was significantly associated with greater anxiety.” (Page 458) |
| Emerson E, et al. (2021)(9) | “For all four PWB indicators, loneliness had a significantly greater association with lower PWB than perceived low social support which, in turn, had a significantly greater association with PWB than social isolation. In the fully adjusted model (Model 3), the association between loneliness and PWB was equivalent to a large effect size for three of the four PWB variable.” (Page 5)  “For all four PWB outcomes, loneliness had a significantly greater association with PWB.” (Page 6) |
| Emerson E, et al. (2021)(10) | “Exposure to loneliness was positively associated with the incidence (GHQ-12) and prevalence (SF-12 Mental) of mental health problems, but not the prevalence of physical health problems (SF-12 Physical). Disability status appeared to moderate the association between loneliness and health, with the difference between the persistent disability and no disability group increasing with exposure to greater levels of loneliness.’ (Page 533)  “Exposure to loneliness was positively associated with the incidence (GHQ-12) and prevalence (SF-12 Mental) of mental health problems, but not the prevalence of physical health problems. (Page 536) |
| Papagavri K, et al. (2020) (42) | “In relation to common mental disorders, the associations were similar in both groups. Those who reported feeling lonely had lower wellbeing, were more likely to have depression, generalised anxiety disorder, agoraphobia, and any type of phobia, in the last 12 months and to report suicidal thoughts in the past week and last year than those who were not lonely. Loneliness was also associated with chronic diseases and poor self- reported health. Analysis of interaction effects found that people with borderline intellectual functioning who reported suicidal thoughts in the last week were more likely to be lonely compared to people who people in the general population who reported suicidal thoughts.” (Page 956) |
| Robinson-Whelen S, et al. (2016)(45) | “As expected, loneliness scores were significantly related to psychological health such that life satisfaction decreased (rZ.42, PP<.001) and depression increased (rZ.29, P<.001) with increasing loneliness, reflecting a medium ES.” (Page 1731)  “Loneliness was significantly related to life satisfaction and depression…(Page 1731)  Even more striking was our finding that loneliness was an independent predictor of life satisfaction and depression even after controlling for demographic and disability characteristics and measures of social integration, and predicted life satisfaction even beyond depressive symptoms.” (Page 1732) |
| Santino N, et al. (2022)(48) | “Significant bivariate correlations were found between … loneliness and life satisfaction (r = –.69, P < .001) (Table 2)” (Page 176) |
| Smith B and Caddick N. (2015)(50) | “However, in this study people’s psychological wellbeing was also damaged due to the intense loneliness that being a body-in-the-world of the care home environment generated. For the participants, loneliness was more than just seclusion or feeling bored. It included a deep felt sense of lost relatedness with others, alienation, and that they do not really belong to themselves anymore.” (Page 4194) |
| Tough H, et al. (2017)(51) | “Loneliness was consistently negatively associated with vitality and mental health in both, partners and persons with SCI, in the unadjusted as well as the adjusted models. Persons with SCI who reported feeling lonely sometimes or often scored on average 15.0% lower in vitality and 14.2% lower in mental health than those who reported never feeling lonely.” (Page 297) |
